# Impact of preoperative 5α-reductase inhibitors on perioperative blood loss in patients with benign prostatic hyperplasia: a meta-analysis of randomized controlled trials

**DOI:** 10.1186/s12894-015-0043-4

**Published:** 2015-06-02

**Authors:** Yi-Ping Zhu, Bo Dai, Hai-Liang Zhang, Guo-hai Shi, Ding-Wei Ye

**Affiliations:** Department of Urology, Fudan University Shanghai Cancer Center, No. 270 Dong an Road, Shanghai, 200032 People’s Republic of China; Department of Oncology, Shanghai Medical College, Fudan University, No. 270 Dong an Road, Shanghai, 200032 People’s Republic of China

**Keywords:** 5α-reductase inhibitor, Benign prostate hyperplasia, Hemorrhage, Meta-analysis

## Abstract

**Background:**

The ability of 5α-reductase inhibitors (5ARIs) to decrease blood loss during transurethral resection of the prostate (TURP) for benign prostatic hyperplasia (BPH) remains controversial. We aimed to conduct a meta-analysis of all randomized controlled trials (RCTs) to establish the role of 5ARI use prior to TURP.

**Methods:**

We searched studies from the electronic databases PubMed, Embase, Scopus, and Cochrane Library from inception to March 25, 2014. Meta-analysis was performed using the statistical software Review Manager version 5.1.

**Results:**

Seventeen RCTs including 1489 patients were examined. We observed that preoperative treatment with finasteride can decrease total blood loss, blood loss per gram of resected prostate tissue, hemoglobin level alteration, microvessel density (MVD), and vascular endothelial growth factor level. Neither finasteride nor dutasteride reduced operative time, prostate volume, or the weight of gland resected. In contrast, pretreatment with dutasteride before TURP did not decrease the total blood loss or MVD.

**Conclusions:**

Pretreatment with finasteride does seem to reduce perioperative blood loss related to TURP for BPH patients. However, the effect of preoperative dutasteride was inconclusive. Further studies are required to strengthen future recommendations regarding the use of 5ARI as a standard pre-TURP treatment and its optimal regimen.

## Background

Transurethral resection of the prostate (TURP) remains the gold standard for patients with benign prostatic hyperplasia (BPH) that failed medical therapy. Perioperative hemorrhage is one of the major complications of TURP, and prolonged bleeding will lead to blood transfusion and clot retention [1]. 5α-Reductase inhibitors (5ARIs), including finasteride and dutasteride, can block the conversion of testosterone to dihydrotestosterone (DHT) and has been used to treat BPH and BPH-related hematuria [2].

Since Hagerty et al. [3] first reported that pretreatment with finasteride appears useful in reducing perioperative bleeding in patients undergoing TURP, emerging studies have reported similar results [4–18]. However, the ability of 5ARI to decrease blood loss during TURP for BPH remains controversial, and several studies have reported no significant benefit of preoperative 5ARIs [19–21]. One systematic review also demonstrated that preoperative finasteride can reduce blood loss during TURP while dutasteride cannot [22]. However, the systematic review was criticized for including a nonrandomized trial [23] and a study comparing photoselective vaporization of the prostate (PVP) instead of TURP with controls [24]. Therefore, we aimed to conduct a meta-analysis of all randomized controlled trials (RCTs) to establish the role of 5ARI use prior to TURP.

## Methods

### Data sources and search strategy

The present meta-analysis was conducted following the Preferred Reporting Items for Systematic Reviews and Meta-Analyses (PRISMA) statement [25].No protocol exists for this meta-analysis. We searched studies from the electronic databases PubMed, Embase, Scopus, and Cochrane Library from inception to March25, 2014.The search terms used were 5α-reductase inhibitor, TURP, transurethral resection of the prostate, 5ARI, BPH, dutasteride, and finasteride. Meanwhile, references from all retrieved papers were manually searched for further relevant articles. We also searched for abstracts of randomized trials from conference proceedings. If the results of the same population were reported more than one time, only the most recent and complete data were included. No language or other restrictions were used in the search.

### Study selection

Studies were considered eligible if they met the following criteria: (1) the study was a RCT, (2) the study participants were BPH patients undergoing monopolar TURP, (3) the main exposure of interest was use of 5ARI in the preoperative period, and (4) the study reported at least one of the following: estimated blood loss(EBL), decrease in hemoglobin (Hb) level, resection weight, blood loss per gram of resected tissue, microvessel density (MVD), and vascular endothelial growth factor (VEGF) level.

We excluded studies if(1) the study was nonrandomized, (2)the full text of the study could not be accessed, (3)outcomes relevant to our interests were not reported, (4) we could not extract data in the appropriate format and failed to obtain the data from the authors, or (5) interventions were bipolar TURP, PVP, or holmium laser enucleation of the prostate (excluded because only one study using PVP and one study using bipolar TURP used 5ARI in the preoperative period, indicating that we could not pool the data into a meta-analysis and perform subgroup analysis because the sample size was too small).

### Data extraction and risk of bias assessment

Data were independently extracted from each study applying a standardized form by two reviewers and then cross-checked. Any disagreement was resolved by discussion between the two authors. If these two authors could not reach a consensus, another author was consulted to resolve the dispute and a final decision was made by a majority vote. The quality of the included RCTs was assessed by the Cochrane Risk of Bias Tool.

### Data synthesis and analysis

We used the mean difference (MD) and relative risk with a 95% confidence interval (CI) for continuous and dichotomous data, respectively. For studies that presented continuous data as median and range values, the means and standard deviations were calculated using statistical algorithms described by Hozo et al. [26].The DerSimonian and Laird random-effects model was used if there was evidence of heterogeneity between the studies, based on the χ^2^ test for heterogeneity and the *I*^2^ test. A *P* value <0.10 and an *I*^2^ value >50%, respectively, were considered high [27]. Otherwise, the fixed-effect model (Mantel-Haenszel) was selected. Publication bias was assessed using inverted funnel plots. Sensitivity analysis was performed to examine whether the effect estimate was robust to exclusion of different criteria. Analysis was performed using the statistical software Review Manager version 5.1.

## Results

### Study characteristics

**Fig. 1:**
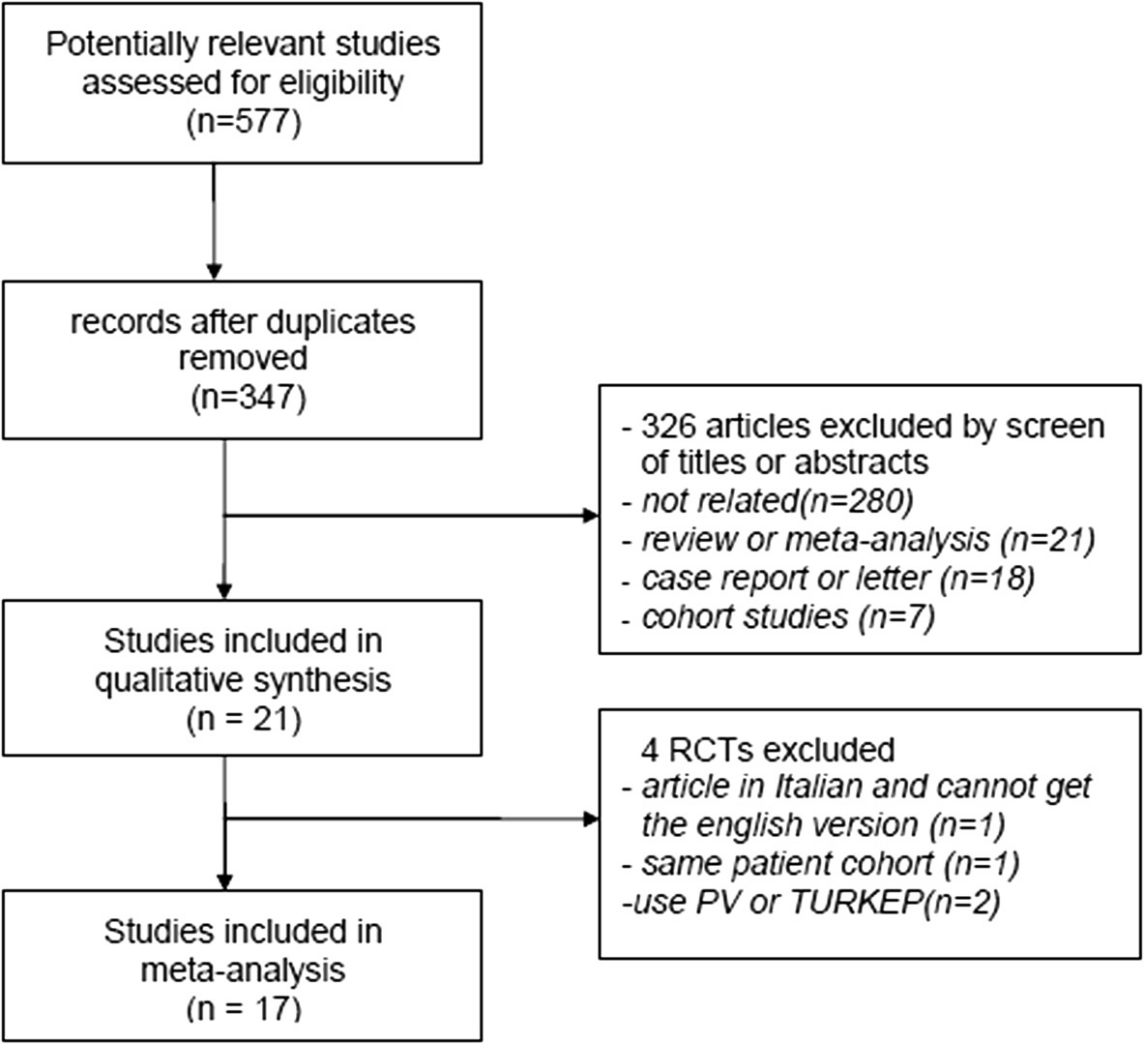
PRISMA flow diagram. PV: Photoselective vaporization of the prostate; RCT: Randomized controlled trial; TUPKEP: Transurethral plasmakinetic enucleation of prostate

Figure 1 traces the flow of our literature search. Briefly, we retrieved 21 potentially relevant studies for quality evaluation and excluded four RCTs [12, 24, 28, 29] for different reasons. One publication [28] was excluded because it was written in Italian, and we could not contact the author for the English version. Two publications were excluded because the interventions were PVP [24] or transurethral plasmakinetic enucleation of prostate [29]. Two publications by Donohue et al. [8, 12] had overlapping populations, and one study was excluded from meta-analysis because it reported a lower number of cases than the one we included [8]. Finally, 17 RCTs [4–11, 13–21] including 1489 patients met the inclusion criteria (746 with 5ARI and 743 without). The characteristics of included RCTs are summarized in Table 1.

**Table 1:** Study characteristics

Study	Country	Age		Sample size		Intervention		Dose and duration	Outcomes evaluated
		5ARI	Control	5ARI	Control	5ARI	Control		
Sandfeldt 2001 [10]	Sweden	69	68	26	29	Fin	placebo	5mg daily, 12 weeks	blood loss, operating time, resection weight, MVD
HäggstrÖm 2002 [13]	Sweden	NM	NM	15	13	Fin	placebo	5mg daily, 12 weeks	VEGF, MVD
Donohue 2002 [8]	UK	69.9	70.2	32	36	Fin	placebo	5mg daily, 2 weeks	blood loss, resection weight
Liu 2003 [14]	China	68.9	68.4	50	50	Fin	blank	5mg daily, 2 weeks	blood loss, Hb alteration, operating time, resection weight, MVD, VEGF
Li 2004 [6]	China	70.7	72.1	40	40	Fin	blank	5mg daily, 1–2 weeks	blood loss, operating time, resection weigh
Özdal 2005 [4]	Turkey	66.9	66.3	20	20	Fin	blank	5mg daily, 4 weeks	blood loss, Hb alteration, resection weight
Lund 2005 [19]	Denmark	66.5	67	16	17	Fin	placebo	5mg daily, 12 weeks	blood loss, operating time, resection weight
Boccon 2005 [16]	France	NM	NM	32	27	Dut	placebo	0.5mg daily,4 weeks	Hb alteration, resection weight
Lekas 2006 [7]	Greece	68.6	68.8	88	90	Fin	blank	5mg daily, 25.3 weeks	blood loss, MVD ,VEGF
Hahn 2007 [20]	multicenter 3-arm study	67/67	66	72/71	70	Dut	placebo	0.5mg daily, 4 weeks before and 2 weeks after TURP	Hb alteration per gram prostate; MVD
Memis 2008 [11]	Turkey	65	64	13	17	Fin	blank	5mg daily, 4 weeks	MVD
Berardinis 2008 [9]	Italy	68	69	100	100	Fin	placebo	5mg twice, 8 weeks	MVD,VEGF
Tuncel 2009 [21]	Turkey	68.1	67.7	27	21	Dut	blank	0.5mg daily,5 weeks	Blood loss, Hb alteration, MVD
Kravchick 2009 [17]	Israel	67.7	66.15	24	22	Dut	blank	0.5mg daily,6 weeks	blood loss, operating time, resection weight
He 2012 [15]	China	64.5	65.5	30	30	Fin	blank	5mg daily, 2 weeks	blood loss, operating time, resection weight, MVD ,VEGF
Pastore 2013 [18]	Italy	65.66	66.7	71	71	Dut	blank	0.5mg daily,6 weeks	blood loss, operating time, resection weight
Liu 2013 [14]	China	69.2	68.4	90	90	Fin	blank	10mg twice, 2 weeks	blood loss, operating time, resection weight, VEGF

*5ARI* 5α-reductase inhibitors, *Fin* finasteride, *Dut* dutasteride, *MVD* microvessel density, *VEGF* vascular endothelial growth factor, *RR* relative risk, *MD* mean difference, *CI* confidence interval

### Risk of bias assessment

The results of the risk of bias assessments are reported in Table 2. Overall, most studies had moderate to high risk of bias. The method of randomization was clearly depicted in only three trials. Allocation concealment was adequately stated in six trials. Blinding was evaluated separately for patients and outcome assessors. Blinding of outcome assessment was part of the trial design in only four studies. All but five trials reported incomplete outcome data.

**Table 2:** Cochrane risk of bias summary of included RCTs

Study	Random Sequence Generation	Allocation Concealment	Blinding of participants and personnel	Blinding of outcome assessment	Incomplete outcome data	Selective outcome reporting	Other sources of bias
Sandfeldt 2001 [10]	Unclear risk	low risk	low risk	low risk	low risk	low risk	low risk
HäggstrÖm 2002 [13]	Unclear risk	Unclear risk	high risk	high risk	low risk	low risk	low risk
Donohue 2002 [8]	Unclear risk	Unclear risk	low risk	Unclear risk	low risk	low risk	low risk
Liu 2003 [5]	Unclear risk	Unclear risk	high risk	high risk	high risk	low risk	low risk
Li 2004 [6]	Unclear risk	Unclear risk	high risk	high risk	high risk	low risk	low risk
Özdal 2005 [4]	Unclear risk	Unclear risk	low risk	Unclear risk	low risk	low risk	low risk
Lund 2005 [19]	low risk	low risk	Unclear risk	Unclear risk	low risk	low risk	low risk
Boccon 2005 [16]	Unclear risk	low risk	low risk	low risk	low risk	low risk	low risk
Lekas 2006 [7]	low risk	Unclear risk	high risk	high risk	low risk	low risk	low risk
Hahn 2007 [20]	Unclear risk	low risk	low risk	low risk	low risk	low risk	low risk
Memis 2008 [11]	Unclear risk	Unclear risk	high risk	high risk	low risk	low risk	low risk
Berardinis 2008 [9]	Unclear risk	low risk	low risk	low risk	low risk	low risk	low risk
Tuncel 2009 [21]	Unclear risk	Unclear risk	high risk	high risk	high risk	low risk	low risk
Kravchick 2009 [17]	high risk	low risk	high risk	high risk	high risk	low risk	low risk
He 2012	Unclear risk	Unclear risk	high risk	high risk	high risk	low risk	low risk
Pastore 2013	low risk	Unclear risk	low risk	Unclear risk	low risk	low risk	low risk
Liu 2013 [14]	Unclear risk	Unclear risk	high risk	high risk	low risk	low risk	low risk

*5ARI* 5α-reductase inhibitors, *Fin* finasteride, *Dut* dutasteride, *MVD* microvessel density, *VEGF* vascular endothelial growth factor, *RR* relative risk, *MD* mean difference, *CI* confidence interval

^a^favors control

### Main outcomes

#### Estimated blood loss

**Fig. 2:**
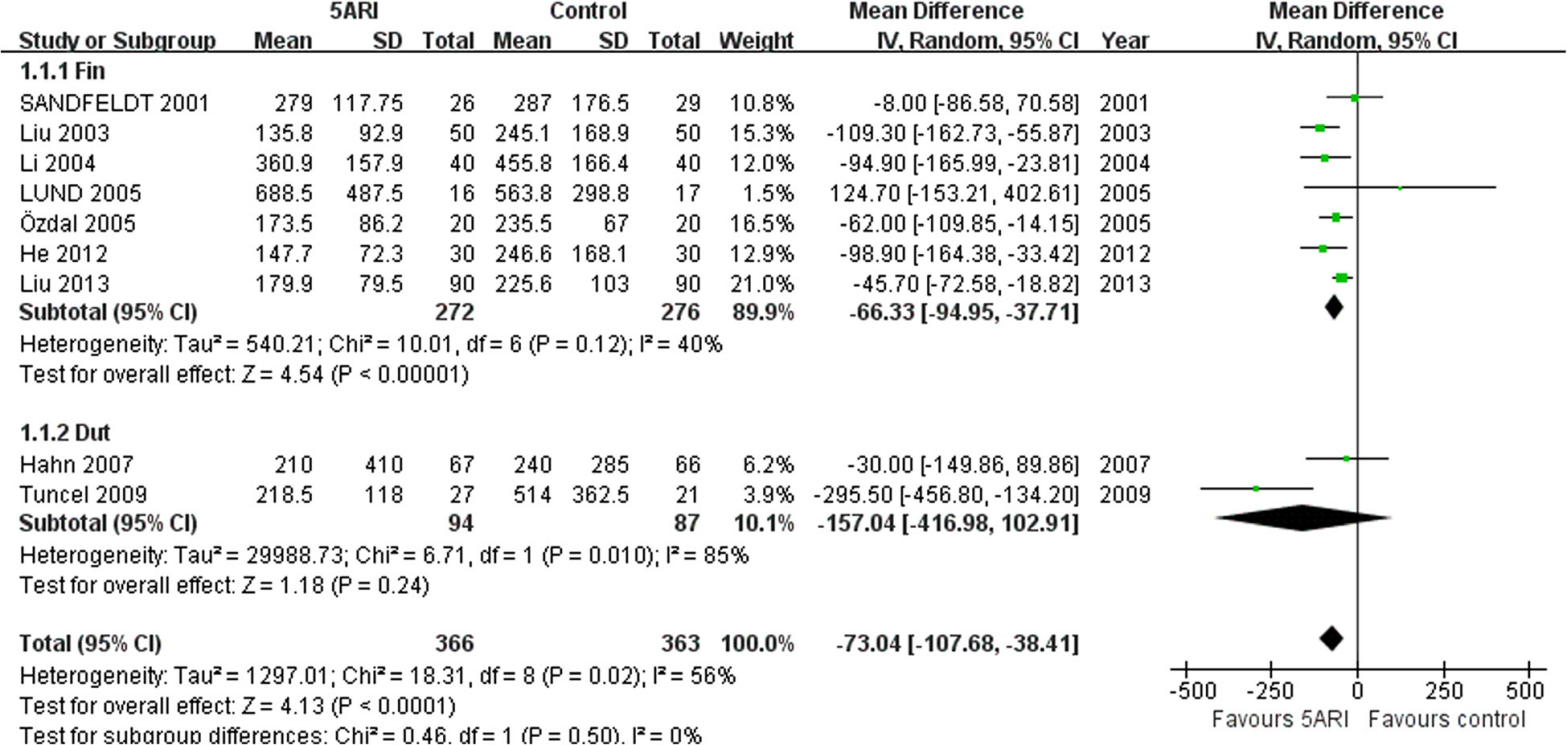
Forest plot presenting the meta-analysis for the effect of 5ARI treatment on blood loss. Pretreatment with finasteride significantly reduced perioperative blood loss (*P* < 0.00001) while dutasteride did not (*P* = 0.24). 5ARI: 5α-Reductase inhibitors; CI: Confidence interval; Dut: Dutasteride; Fin:Finasteride

Nine RCTs including 729 patients evaluated EBL between a 5ARI group and a control group (including seven RCTs for finasteride and two RCTs for dutasteride). Pooling data showed a significant benefit of 5ARI on reducing EBL in the finasteride group, whereas no conspicuous difference was observed in the dutasteride subgroup. The random-effects model was reported because there was evidence of significant heterogeneity (Fig. 2).

#### Blood loss per gram of resected prostate tissue

**Fig. 3:**
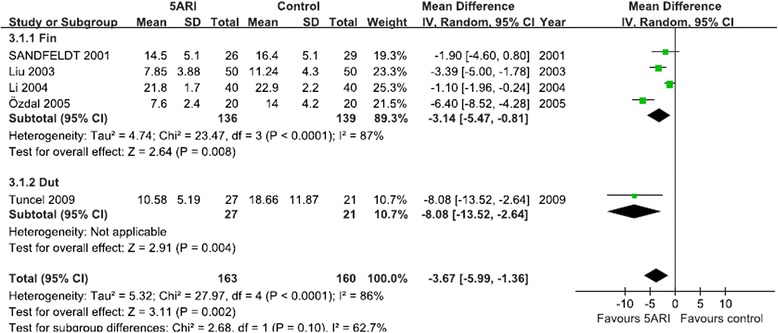
Forest plot presenting the effect of 5ARI treatment on blood loss per gram of resected prostate tissue

Five RCTs that included 323 patients evaluated blood loss per gram of resected prostate tissue between 5ARI and control groups (including four RCTs for finasteride and one RCT for dutasteride). Pooling data showed a significant benefit of 5ARI on reducing blood loss per gram of resected prostate tissue in both the finasteride and dutasteride groups. The random-effects model was reported because there was evidence of significant heterogeneity (Fig. 3).

#### Hb alteration

**Fig. 4:**
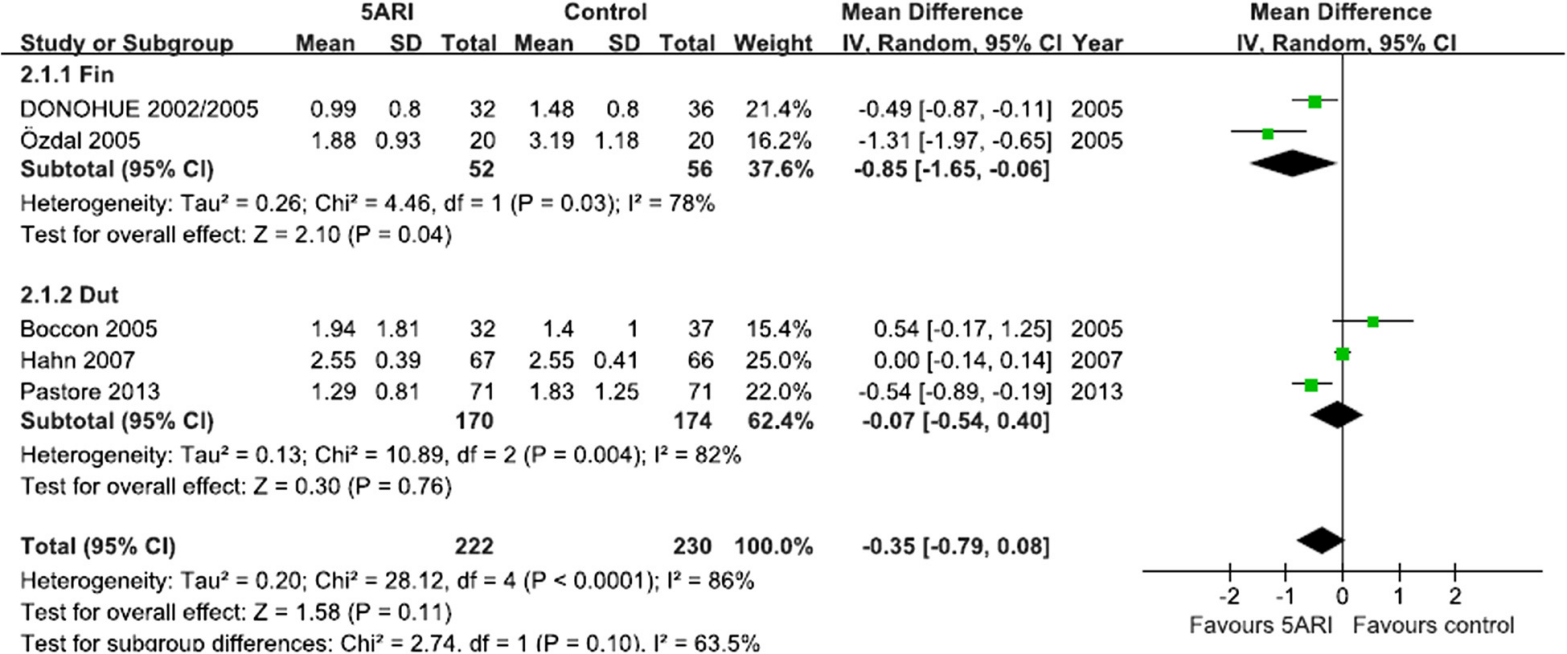
Forest plot presenting the effect of 5ARI treatment on Hb change before and after TURP

Five RCTs including 452patients reported Hb change before and after TURP (including two RCTs for finasteride and three RCTs for dutasteride). When pooled, the results showed that 5ARI reduced the Hb change in the finasteride group but not in the dutasteride group. The random-effects model was selected because there was evidence of significant heterogeneity (Fig. 4).

#### Blood transfusions needed

**Fig. 5:**
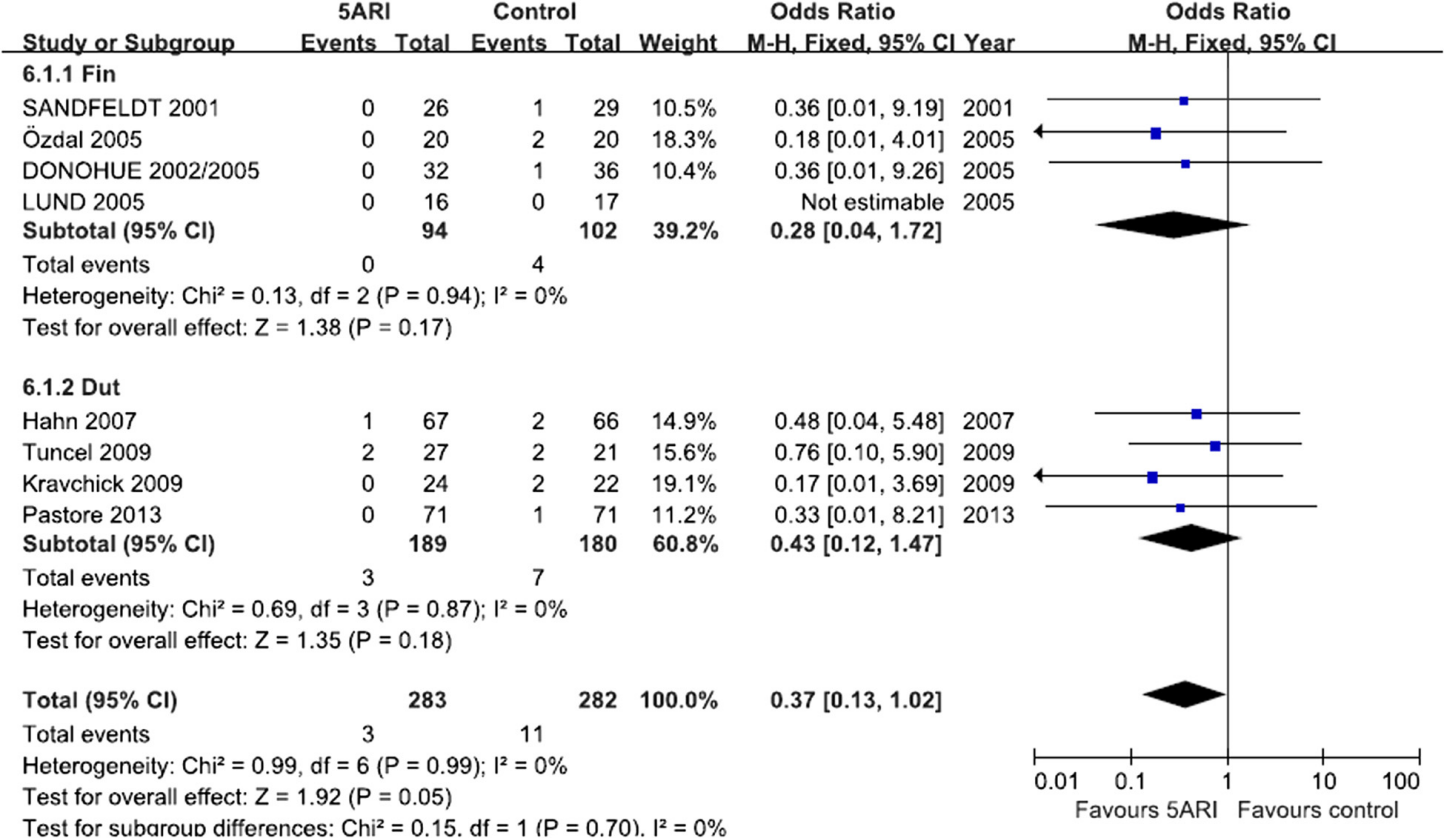
Forest plot presenting the effect of 5ARI treatment on Blood transfusion needed

Eight RCTs including 565 cases evaluated patients who needed a blood transfusion (including four RCTs for finasteride and four RCTs for dutasteride). When pooled, although there was a trend in favor of the 5ARI group, the result did not show significant differences between treatment and control groups (*P* = 0.05). According to our analysis, no heterogeneity was found among the trials (*I*^2^ = 0); thus, a fixed-effects model was chosen for the analysis (Fig. 5).

#### MVD and VEGF expression after 5ARI treatment

**Fig. 6:**
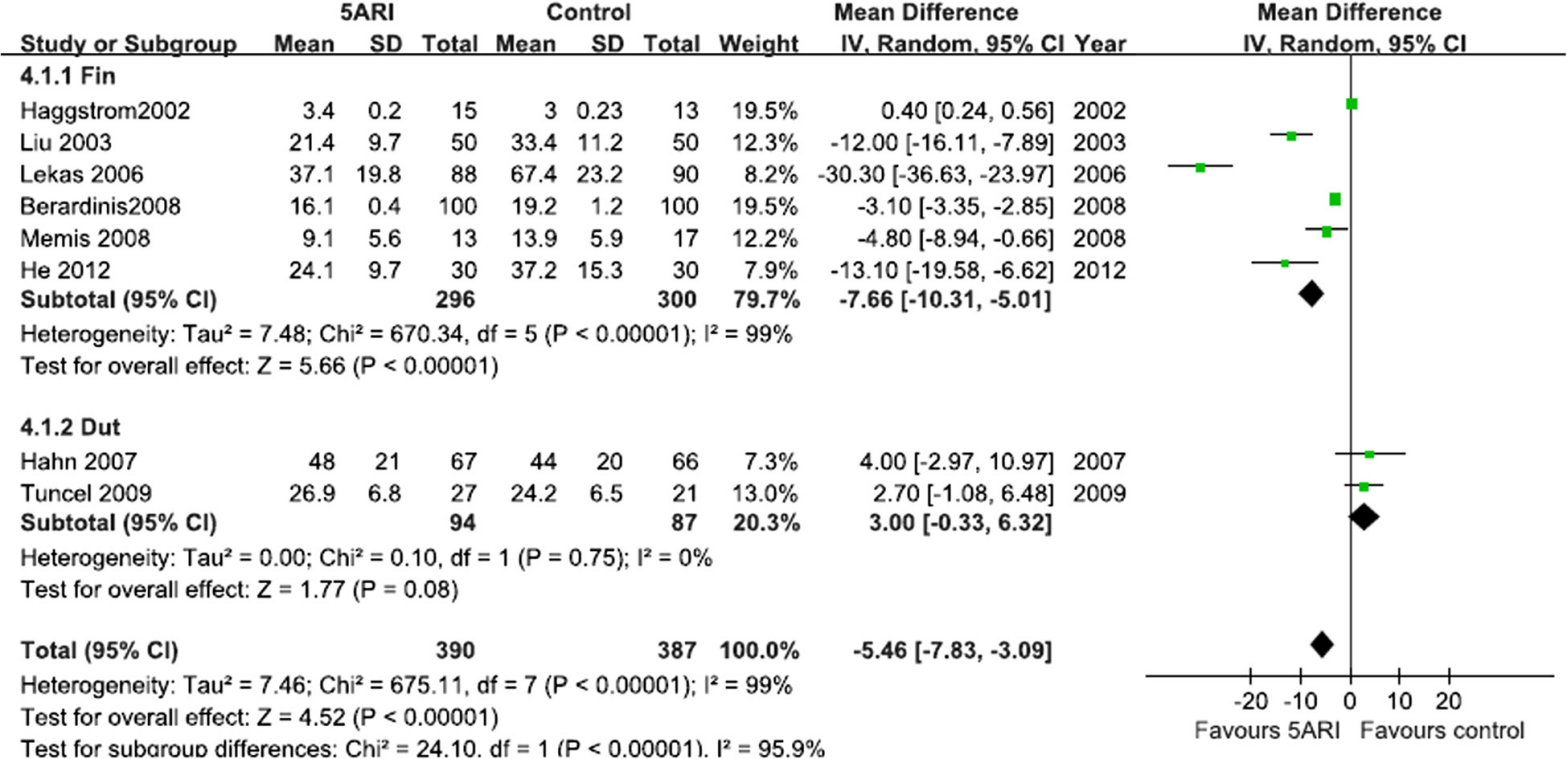
Forest plot presenting the effect of 5ARI treatment on MVD

**Fig. 7:**
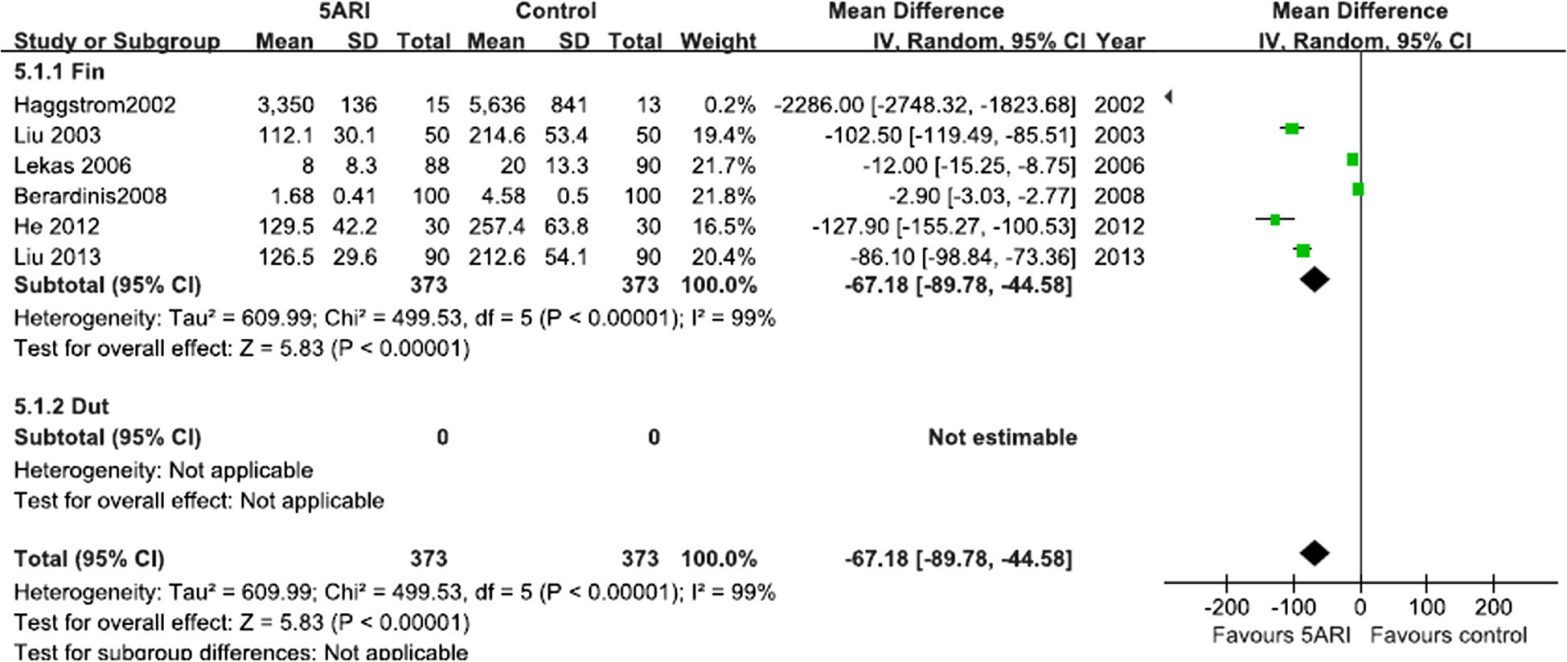
Forest plot presenting the effect of 5ARI treatment on VEGF

To elucidate the mechanism of 5ARI action, we identified eight RCTs that evaluated MVD (including six RCTs for finasteride and two RCTs for dutasteride), and six RCTs evaluated VEGF expression after 5ARI treatment (six RCTs including 746 patients for finasteride).The overall result of the meta-analysis showed that the MVD and VEGF of the resected prostate tissue were lower in the finasteride group than in the control group, whereas oral dutasteride did not decrease MVD. The random-effects model was reported because there was evidence of significant heterogeneity (Figs. 6 and 7).

#### Other parameters

We also evaluated other parameters between the 5ARI and control groups in the present meta-analysis, including operative time, weight of gland resected, and prostate volume. The pooled data showed that both finasteride and dutasteride did not reduce operative time, prostate volume, or weight of gland resected. On the contrary, lesser gland tissue was resected and the prostate volume was smaller in the control group (Table 3).

**Table 3:** Study outcomes comparing 5ARI with control

Outcomes	No of studies (Fin/Dut)	No. of patients		RR/MD (Total)	95%CI (Total)	P value (Fin/Dut/Total)	Heterogeneity (Total)			
		5ARI(Fin/Dut)	Control(Fin/Dut)				chi^2^	df	I^2^%	P value
Blood loss	7/2	272/94	276/87	−73.04	−107.68,-38.41	<0.00001/0.24/<0.0001	18.31	8	56	0.27
Hb alteration	2/3	52/170	56/174	−0.35	−0.79,0.08	0.04/0.76/0.11	28.12	4	86	<0.0001
Blood loss/g tissue	4/1	136/27	139/21	−3.67	−5.99,-1.36	0.008/0.004/0.002	27.97	4	86	<0.0001
MVD	6/2	296/94	300/87	−5.46	−7.83,-3.09	<0.00001/0.08/<0.00001	675.11	7	99	<0.00001
VEGF	6/0	373/0	373/0	−67.18	−89.78,-44.58	<0.00001	499.53	5	99	<0.00001
Operative time	6/4	252/189	256/180	−3.96	−8.17,2.87	0.12/0.35/0.07	32.46	9	72	0.0002
Transfusion needed	4/4	94/189	102/180	0.37	0.13,1.02	0.17/0.18/0.05	0.99	6	0	0.99
Gland resected	7/4	254/189	262/180	1.09	0.3,1.87	0.01^a^/0.4/0.006	8.46	10	0	0.58
Prostate volume	6/2	291/98	297/92	1.85	0.60,3.10	0.003^a^/1.00/0.004	4.33	7	0	0.74

### Sensitivity analysis and publication bias

**Fig. 8:**
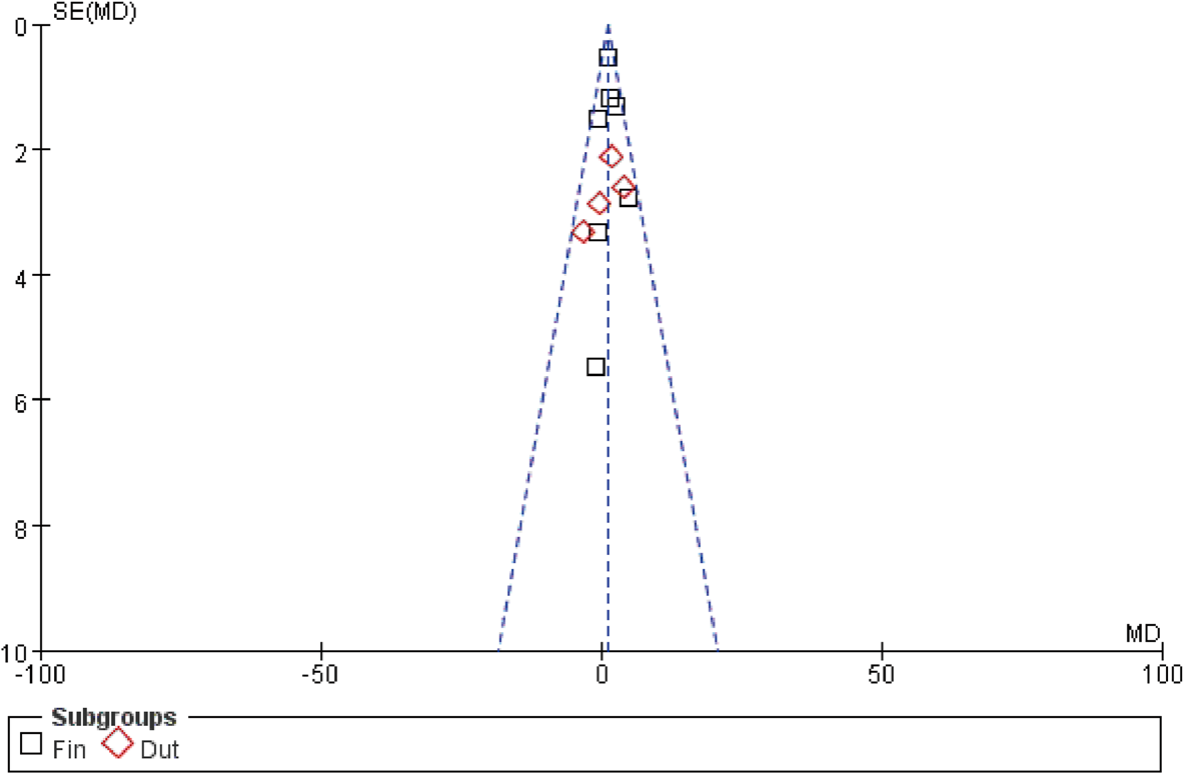
Funnel plot of the studies represented in our meta-analysis. This funnel plot provided us with a qualitative estimation of publication bias of the studies, and no evidence of bias was found. Dut: Dutasteride; Fin:Finasteride

Sensitivity analysis was performed by sequential removal of individual studies and cumulative statistics for all comparisons of all subjects. The pooled MD was not influenced by the result of any individual study. Funnel plots were used to assess the publication bias. All studies lie inside the 95% CIs, with an even distribution around the vertical, indicating no obvious publication bias (Fig. 8).

## Discussion

5ARI is commonly used for treating BPH and hematuria of prostatic origin. However, the concept of preoperatively administering 5ARI to reduce blood loss during TURP has not been accepted by most urologists. In a United Kingdom-based survey, although 98% of urologists used finasteride for hematuria of prostatic origin, only 4% used it before TURP [30]. In the present meta-analysis involving 17 RCTs and 1489 participants, we demonstrated that preoperative treatment with finasteride for 2 weeks to 6 months could decrease blood loss during TURP for BPH. In contrast, pretreatment with dutasteride before TURP did not change the total blood loss.

Testosterone is a stimulator of VEGF, and androgen deprivation leads to decreased blood flow in the prostate [31, 32]. Finasteride blocks the conversion of testosterone to DHT, resulting in decreased activity of the androgen-controlled growth factors, such as VEGF. MVD is another histologic indicator of angiogenesis in BPH patients. Emerging data have shown that finasteride treatment prior to TURP significantly decreased MVD in the prostate tissue [9–13]. Our meta-analysis confirmed the results of previous studies, demonstrating that finasteride could significantly decrease MVD and VEGF of the prostate tissue compared with controls.

Finasteride, a type II 5ARI, has been shown to decrease the size of the prostate, and therefore the operative time might also be decreased secondary to the smaller gland. However, the present meta-analysis showed that neither finasteride nor dutasteride prior to TURP reduced operative time, prostate volume, or weight of the gland resected. One possible explanation is that a decrease in the size of the prostate gland requires up to 6 months of finasteride to occur [33]. However, most RCTs in our meta-analysis used finasteride ranging from 2 to 12 weeks, not enough for shrinkage of the prostate gland. On the contrary, the effect of finasteride on hematuria was more rapid than may reasonably be attributed to decreased prostate size. Liu et al. [5] showed that MVD and VEGF decreased obviously in patients treated with finasteride for 14 days. Taken together, the mechanism by which finasteride decreased blood loss during TURP was probably related to decreased vascularity in the prostate rather than to a smaller prostate and shorter operative time.

Dutasteride, a dual 5ARI, provides greater suppression of 5α-reductase because it antagonizes both type I and II receptors [34]. In theory, it should produce an effect that is better than, or at least similar to, finasteride. However, in our meta-analysis, we did not find any differences between the dutasteride and control groups with regard to EBL, decrease in Hb, resection weight, prostate volume, transfusions needed, and operative time. Only one RCT with limited cases showed that pretreatment with dutasteride could decrease blood loss per gram of resected tissue [21].To our surprise, unlike finasteride, pooling data of two RCTs including 181 cases showed that dutasteride treatment did not decrease MVD, which might partially explain why dutasteride was not effective in reducing EBL. In addition, because dutasteride is a newer drug, the patient populations recruited to these RCTs were certainly different from the cohorts that were available for the finasteride RCTs. Thus, selection bias maybe another plausible explanation for the failure to find a difference in the dutasteride group. The exact reason dutasteride was not effective in reducing EBL and MVD remains unclear, and additional well-designed RCTs are needed to establish its actual role.

Because approximately 50% of the variability in blood loss can be accounted for by the amount of resected tissue in TURP, the resection weight and/or prostate size should be taken into account when calculating EBL [35]. In one study by Sandfeldt et al. [10], a positive correlation between EBL and resection weight was reported. According to Hagerty et al. [3], patients with resected weights of >30 g who received finasteride before TURP needed fewer blood transfusions than those who did not receive the drug. Although we found no differences between 5ARI and control groups with regard to transfusions needed, it may be that the trial sample sizes were not large enough to generate enough data for detecting significant effects. In addition, most RCTs in our meta-analysis did not perform subgroup analysis based on resection weight (e.g.,>30g) or prostate size. Further studies are required to fully assess the hypothesis that the benefit of 5ARI treatment would manifest at resected weights of >30 or >40 g or set a cutoff value for prostate size.

The present meta-analysis had some limitations. First was the quality of the studies assessed; most of the included RCTs did not describe randomization concealment and blinding techniques. Second was the substantial heterogeneity among studies, which was probably caused by the variability among oral 5ARI regimens and outcome measurements. Oral 5ARI regimens were not standardized, and the studies varied in the dose of 5ARI used as well as the drug duration and frequency. Data were therefore analyzed using a random-effects model, which accounts for both within-study and between-study variability. Finally, inherent in any meta-analysis is the possibility of publication bias; that is, small studies with null results tend not to be published. However, several RCTs included in the meta-analysis also contained negative results [11, 19–21], and the funnel plot did not provide any evidence of publication bias.

## Conclusion

Pretreatment with finasteride does seem to reduce perioperative blood loss related to TURP for BPH patients. This effect was probably due to decreased vascularity in the prostate rather than a smaller prostate or shorter operative time. However, the effect of preoperative dutasteride was inconclusive. Further studies are required to strengthen future recommendations regarding the use of 5ARI as a standard pre-TURP treatment and its optimal regimen.

## Abbreviations

5ARI: 5α-Reductase inhibitor
BPH: Benign prostatic hyperplasia
CI: Confidence interval
DHT: Dihydrotestosterone
EBL: Estimated blood loss
Hb: Hemoglobin
MD: Mean difference
MVD: Microvessel density
PVP: Photoselective vaporization of the prostate
RCT: Randomized controlled trial
TURP: Transurethral resection of the prostate
VEGF: Vascular endothelial growth factor.
